# The Receptivity to Safety-Related Mobile Apps Among Commercial Fishing Captains: Descriptive Exploratory Study

**DOI:** 10.2196/33638

**Published:** 2022-11-08

**Authors:** Maria T Bulzacchelli, Jenna M Bellantoni, Leigh McCue, Jerry Dzugan

**Affiliations:** 1 Krieger School of Arts and Sciences Johns Hopkins University Baltimore, MD United States; 2 Department of Mechanical Engineering George Mason University Fairfax, VA United States; 3 Alaska Marine Safety Education Association Sitka, AK United States

**Keywords:** mobile app, mobile device, mobile phone, smartphone, safety, workplace safety, occupational safety, mobile health, mHealth, commercial fishing, cross-sectional study

## Abstract

**Background:**

Mobile apps addressing a variety of workplace safety issues have proliferated over the last decade as mobile technology has advanced and smartphone ownership has increased. Workplace safety interventions are often designed for a specific work site. However, some of the most dangerous jobs are ones in which workers frequently change field locations, such as commercial fishing. Mobile apps may be particularly suitable for delivering safety interventions to these workers.

**Objective:**

We sought to gauge the potential for using mobile apps to deliver safety interventions to commercial fishing workers. The purpose of this paper is to describe how fishermen use their mobile devices during fishing operations and identify any mobile apps they already use for safety.

**Methods:**

Participants comprised commercial fishing captains who already owned an iOS or Android smartphone or tablet. They completed a questionnaire that asked about their current mobile device use and their use of safety-related mobile apps, in addition to questions about their fishing operations. We performed descriptive analyses of the data.

**Results:**

A total of 61 participants completed the questionnaire. The most common types of mobile devices participants reported owning were iPhones (n=36, 59%) and Android phones (n=24, 39%). Most participants (n=53, 87%) reported using their mobile device for both work and personal purposes, including while out at sea (n=52, 85%). Over half of the participants reported that they had either safety-related apps (n=17, 28%) or apps that help them with their work (n=35, 57%). The types of apps most frequently mentioned were apps for weather, wind, tides, and navigation.

**Conclusions:**

The results of this study indicate that some commercial fishing captains who own a mobile device are receptive to using safety-related apps for work. Apps that help avoid hazards by monitoring environmental conditions and apps optimized for use on smartphones may be most likely to be adopted and used. Overall, these results suggest that mobile apps are a promising avenue for improving safety among workers in commercial fishing and similar occupations.

## Introduction

In the past 15 years, smartphone ownership has grown dramatically. According to the Pew Research Center, 85% of adults in the United States owned a smartphone in 2021, compared to 70% in 2016 and 35% in 2011 [1]. With the rapid growth of mobile technology and the resulting ubiquity of smartphones, health and safety interventions delivered through mobile apps have proliferated [2]. There are now tens of thousands of mobile health (mHealth) apps providing reference information, training and education, monitoring, clinical score calculators, reminders, and communication functions [3,4].

One area with potential for greatly expanding the reach of mHealth interventions is workplace safety. A total of 91% of full-time workers use a smartphone at work [5]. Technology that integrates interventions into a small device that almost all workers already carry with them opens up numerous possibilities for promoting safety and preventing injuries. Industries and occupations in which workers frequently change field locations, such as construction, tree care, commercial fishing, as well as certain installation, maintenance, and repair occupations, may be especially promising areas for mHealth interventions that can be accessed wherever workers are. The National Institute for Occupational Safety and Health has developed mobile apps to address a variety of occupational hazards including a ladder safety app, a heat safety tool, a sound level meter, a personal protective equipment (PPE) inventory tracker, and multiple apps addressing aspects of ergonomics [6]. Dozens of other occupational safety and health apps are available on the app market as well [7].

Little is known about how workers use occupational safety apps or even how they use their mobile devices in the workplace. Studies that have examined workers’ use of mobile devices or apps relevant to various work settings have produced mixed findings. In a scoping review that looked at nurses’ use of personal smartphones in clinical settings, de Jong et al [8] found that nurses frequently used their personal smartphones to look up medical information and communicate with others on the patient-care team. In a mixed methods study, Patel et al [9] gave 16 physicians access to a mobile clinical decision support tool; 9 of the 16 doctors used the app during the study period while 7 did not. They found that 5 of the doctors accounted for 90% of the interactions with the app, and just 2 accounted for 56% of the interactions with the app over the study period. Snipes et al [10] pilot tested a mobile app to promote PPE use among farmworkers. Some participants reported technical problems with the phone such as low battery or the screen freezing, but 73% encountered no barriers to using the phone. For consistency, participants were given a particular type of phone to use for the pilot testing and they were also given specific types of PPE, but, notably, 75% of participants already had a personal mobile phone at baseline and 39% of those workers used it always or sometimes. Studies of apps that monitor UV conditions and provide tailored sun protection advice and warnings have found apps to be effective in improving some sun safety behaviors among participants from the general population, but not all participants used the apps [11,12]. These studies suggest that many workers already use their smartphones for work purposes, but even when asked to try apps designed for use in specific work settings, only a portion of workers may use the apps.

Commercial fishing consistently ranks as one of the most hazardous occupations in the United States. From 2000 to 2015, fishers and related fishing workers had a fatal injury rate of 117 per 100,000 full-time equivalent workers, approximately 29 times the all-worker rate [13]. Mobile apps should be explored as low- or no-cost interventions to protect this worker population from injury and death. A first step is determining how receptive commercial fishing workers would be to such apps. Technology adoption theories suggest that experienced mobile device users are more likely to adopt new mobile apps than individuals with little experience using mobile devices [14]. Little is known about how commercial fishing workers use mobile devices, especially when out at sea. The purpose of this paper is to describe how fishermen use their mobile devices during fishing operations and identify any mobile apps they already use for safety.

## Methods

### Study Population

Participants were a convenience sample of commercial fishing vessel captains recruited for a study field-testing 2 mobile apps that aim to address certain safety issues related to fishing operations [15]. To be eligible for our study, participants had to be commercial fishing captains who already owned an iOS or Android smartphone or tablet. Participant recruitment and data collection began in July 2018 and were completed in October 2020. A detailed description of our recruitment and data collection procedures is published elsewhere [15].

### Data Collection and Key Variables

Participants completed a baseline questionnaire that was either administered by a researcher in person or self-administered online. The questionnaire asked about participants’ commercial fishing operations and experience, some of their safety practices and concerns about safety, their current mobile device use, and their use of safety-related mobile apps (Textbox 1). Prior to data collection, a member of the study team with extensive commercial fishing experience (JD) reviewed all questionnaire items to ensure their suitability for this population. The items about mobile device use and safety-related app use were exploratory in nature and left open to each participant’s interpretation.

**Questionnaire items about participants’ mobile device and app use.**

What kinds of mobile devices do you have, specifically?

- Do you have an iPhone? (Yes/no)
- Do you have an iPad? (Yes/no)
- Do you have an iPod Touch? (Yes/no)
- Do you have an Android phone? (Yes/no)
- Do you have an Android tablet? (Yes/no)

Thinking about your [device]… Do you use your [device] for work, personal use, or both? (Work only/personal use only/both work and personal use)

Do you ever use your [device] when you are out at sea? (Yes/no)

On your [device], do you have any apps that help you with your commercial fishing work? (Yes/no; if yes: which ones?)

On your [device], do you have any safety apps or apps that you use for the purpose of making you safer, either at work or on your own time? (Yes/no; if yes: which ones?)

### Data Analysis

We performed descriptive analyses of key variables, computing mean values for numeric variables and frequencies for categorical variables. We excluded 2 participants from the calculations of mean and median years of experience because both of these participants gave implausible values, reporting greater years of experience working as a commercial fishing captain than years of experience working on commercial fishing vessels generally. We summarized the free-text responses into broad categories by manually performing a content analysis.

### Ethics Approval

This study was approved by the Johns Hopkins University Homewood Institutional Review Board (PR00015355 HIRB00011417).

## Results

A total of 61 commercial fishing captains participated in our study and completed the baseline questionnaire. The mean age of our sample was 47.3 (SD 14.4) years, with a median of 47 (IQR 34-56) years. Participants had an average of 27.1 years of experience working on commercial fishing vessels and an average of 19.4 years of experience as a commercial fishing captain (Table 1). A total of 57 (93%) participants identified as male.

Per the study’s eligibility criteria, all participants owned at least one mobile device. When asked what kinds of mobile devices they owned, the largest proportion reported owning an iPhone (n=36, 59%), followed by an Android phone, iPad, Android tablet, and iPod Touch (Table 2). Most participants (n=53, 87%) reported using their mobile device for both work and personal use. A similar proportion indicated that they take at least one of their devices with them out at sea. Just over half of the participants (n=35, 57%) indicated they have apps that help them with commercial fishing work, and 28% (n=17) indicated they have safety-related apps. Participants who reported having apps for commercial fishing work or safety most frequently named apps for weather, wind, tides, and navigation, including apps that combine these functions.

**Table 1:** Participants’ age and years of experience (N=61).

Characteristic	Mean (SD)	Median (IQR)
Age	47.3 (14.4)	47 (34-56)
Years of experience working on commercial fishing vessels^a^	27.1 (13.3)	30 (15-37.5)
Years of experience working as a commercial fishing vessel captain^a^	19.4 (13.9)	18 (6.5-29.5)

^a^Two participants were excluded: n=59.

**Table 2:** Mobile device use among participants (N=61).

Variable			Participants, n (%)
**Mobile device ownership^a^**			
	iPhone	36 (59)	
	Android phone	24 (39)	
	iPad	14 (23)	
	Android tablet	2 (3)	
	iPod Touch	2 (3)	
**Mobile device use**			
	Use for both work and personal use	53 (87)	
	Use when out at sea	52 (85)	
**Mobile app use**			
	Use apps for commercial fishing work	35 (57)	
	Use safety apps	17 (28)	

^a^Categories are not mutually exclusive; participants could select all that apply.

## Discussion

### Principal Results and Implications

This study demonstrates that approximately 85% of commercial fishing captains who own an iOS or Android device use it for both work and personal use, including while at sea. Additionally, most captains already use mobile apps for commercial fishing work or safety purposes, most commonly apps for weather, wind, tides, and navigation. All but 1 participant reported owning a smartphone, whereas only about one-quarter reported owning a tablet computer.

These results suggest that commercial fishing captains may be receptive to additional safety-related mobile apps, especially apps that provide real-time information about vessel location and environmental conditions. Apps that monitor changing conditions and provide actionable information to help avoid hazards may be particularly useful to this population. Expanding some popular existing apps to incorporate additional safety functions could be a relatively easy way to increase the availability and uptake of mobile safety interventions. In addition, mobile apps aimed at protecting workers in commercial fishing and similar occupations may be more readily adopted if they are optimized for use on smartphones.

### Limitations

An important limitation of this study is that our sample only included captains who already owned a smartphone or tablet and, therefore, are probably more inclined to adopt mobile technologies than those who do not already have a mobile device. On the one hand, this limitation may lead us to overestimate the potential reach of mobile interventions for commercial fishing safety. On the other hand, our findings are useful for informing the development of mobile apps targeting the specific population most likely to adopt and benefit from them.

While we do not know what proportion of all commercial fishing captains own a smartphone or tablet, our sample appears to be representative of this worker population in at least one important way. The median age of participants in this study (47 years) is very similar to the median age of workers in the agriculture, forestry, fishing, and hunting industry sectors nationally (47.8 years in 2019) [16]. This is relevant for assessing this population’s receptivity to mobile interventions because workers in this industry tend to be older than workers in other industries, and smartphone ownership is related to age. The median age of all employed persons in the United States was 42.3 years in 2019 [16]. In 2021, smartphone ownership in the country was 95% among those aged 30 to 49 years but only 83% among those who are 50 to 64 years old [1].

Another limitation of this study is that we asked participants if they have safety apps on their mobile device, but we did not ask participants how frequently they use the apps mentioned and why. It is possible to have apps on a device that are rarely used, which would reduce the amount of protection afforded by the apps. Additional research is needed to determine factors that influence the actual use of safety apps among this worker population and any barriers to using the apps they might experience.

### Conclusion

The results of this study indicate that some commercial fishing captains who own a mobile device are receptive to using safety-related apps for work. Apps that help avoid hazards by monitoring environmental conditions may be especially appealing to this population, and apps that are optimized for use on smartphones may be most likely to be adopted and used. Overall, these results suggest that mobile apps are a promising avenue for improving safety among commercial fishing workers.

## Abbreviations

mHealth: mobile health
PPE: personal protective equipment

## Data Availability

The data sets generated and analyzed in this study are not publicly available due to participant confidentiality.
